# Management of patients with high-risk pulmonary embolism: a narrative review

**DOI:** 10.1186/s40560-018-0286-8

**Published:** 2018-03-02

**Authors:** Takeshi Yamamoto

**Affiliations:** Division of Cardiovascular Intensive Care, Nippon Medical School Hospital, 1-1-5 Sendagi, Bunkyo-ku, Tokyo, 113-8603 Japan

**Keywords:** Pulmonary embolism, Multidisciplinary management, Thrombolytic therapy, Catheter-directed treatment, Surgical embolectomy

## Abstract

High-risk pulmonary embolism (PE) is a life-threatening disorder associated with high mortality and morbidity. Most deaths in patients with shock occur within the first few hours after presentation, and rapid diagnosis and treatment is therefore essential to save patients’ lives. The main manifestations of major PE are acute right ventricular (RV) failure and hypoxia. RV pressure overload is predominantly related to the interaction between the mechanical pulmonary vascular obstruction and the underlying cardiopulmonary status. Computed tomography angiography allows not only adequate visualization of the pulmonary thromboemboli down to at least the segmental level but also RV enlargement as an indicator of RV dysfunction. Bedside echocardiography is an acceptable alternative under such circumstances. Although it does not usually provide a definitive diagnosis or exclude pulmonary embolism, echocardiography can confirm or exclude severe RV pressure overload and dysfunction. Extracorporeal membrane oxygenation support can be an effective procedure in patients with PE-induced circulatory collapse. Thrombolysis is generally accepted in unstable patients with high-risk PE; however, thrombolytic agents cannot be fully administered to patients with a high risk of bleeding. Conversely, catheter-directed treatment is an optimal treatment strategy for patients with high-risk PE who have contraindications for thrombolysis and is a minimally invasive alternative to surgical embolectomy. It can be performed with a minimum dose of thrombolytic agents or without, and it can be combined with various procedures including catheter fragmentation or embolectomy in accordance with the extent of the thrombus on a pulmonary angiogram. Hybrid catheter-directed treatment can reduce a rapid heart rate and high pulmonary artery pressure and can improve the gas exchange indices and outcomes. Surgical embolectomy is also performed in patients with contraindications for or an inadequate response to thrombolysis. Large hospitals having an intensive care unit should preemptively establish diagnostic and therapeutic protocols and rehearse multidisciplinary management for patients with high-risk PE. Coordination with a skilled team comprising intensivists, cardiologists, cardiac surgeons, radiologists, and other specialists is crucial to maximize success.

## Background

High-risk pulmonary embolism (PE), which presents as shock or persistent hypotension, is a life-threatening disorder associated with high mortality and morbidity [1–3]. The 30-day mortality rate of patients with PE who develop shock ranges from 16 to 25% and that of patients with cardiac arrest ranges from 52 to 65% [4, 5]. Most deaths in patients presenting with shock occur within the first hour after presentation [6]; therefore, rapid therapeutic action is essential to save patients’ lives. PE is caused by abrupt obstruction of pulmonary arteries by thrombi that have mostly formed in the deep veins of the lower limbs or pelvis in more than 90% of affected patients. It is estimated that nearly half of PEs occur in a hospital or health care-related institution [4, 7, 8]. Hospitalized critically ill patients are at high risk for PE [9, 10]. The management of PE in a critically ill patient admitted to the intensive care unit can be exceedingly complex [11]. Intensivists should know how to appropriately care for patients with high-risk PE of both in-hospital onset and out-of-hospital onset [12, 13]. The present review critically assesses data that have contributed to substantial improvement in the management strategies for high-risk PE in recent years.

## Pathophysiology

### Circulatory failure

**Fig. 1:**
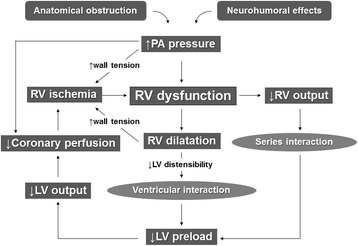
Pathophysiologic cycle of high-risk PE. PE pulmonary embolism, PA pulmonary artery, RV right ventricular, LV left ventricular

The main manifestations of major PE are acute right ventricular (RV) failure and hypoxia. RV pressure overload is predominantly related to the interaction between the mechanical pulmonary vascular obstruction and the underlying cardiopulmonary status. Additional factors of pulmonary vasoconstriction include neural reflexes, the release of humoral factors from platelets (i.e., serotonin and platelet-activating factor), plasma (i.e., thrombin and vasoactive peptides C3a, C5a), tissue (i.e., histamine), and systemic arterial hypoxia, all of which are associated with increased RV afterload [14]. Heart failure induced by major PE results from a combination of increased wall stress and cardiac ischemia, which compromise RV function and impair left ventricular (LV) output in multiple interactions (Fig. 1) [2]. With increasing RV load and wall stress, RV systolic function becomes depressed and cardiac output begins to decrease. The LV preload consequently decreases because the ventricles are aligned in series. LV preload is additionally impaired by decreased LV distensibility as a consequence of a leftward shift of the interventricular septum and of pericardial restraint, both of which are related to the degree of RV dilatation [15, 16]. A further decrease in LV flow results in systemic hypotension. Decreases in the mean arterial pressure associated with increases in the RV end-diastolic pressure impair the subendocardial perfusion and oxygen supply [17]. Increased oxygen demands associated with elevated wall stress coupled with the decreased oxygen supply have been shown to precipitate RV ischemia, which is thought to be the cause of RV failure. Clinical evidence of RV infarction as a consequence of the preceding condition has been demonstrated in patients with and without obstructive coronary disease.

The mean pulmonary arterial pressure that can be generated by the right ventricle is 40 mmHg in individuals without cardiopulmonary disease [18]. Therefore, when the pulmonary arterial pressure exceeds 40 mmHg during the acute phase of PE, physicians should suspect recurrent PE or chronic thromboembolic pulmonary hypertension.

### Respiratory failure

Gas exchange abnormalities in patients with PE are complex and related to the size and characteristics of the embolic material, the extent of the occlusion, the underlying cardiopulmonary status, and the length of time since embolization [2]. Hypoxia has been attributed to an increase in alveolar dead space, right-to-left shunting, ventilation–perfusion mismatch, and a low mixed venous oxygen level [2, 19, 20]. The two latter mechanisms are proposed to account for most cases of observed hypoxia and hypocapnia before and after treatment. Zones of reduced flow in obstructed vessels combined with zones of overflow in the capillary bed served by unobstructed vessels result in ventilation–perfusion mismatch, which contributes to hypoxia. In addition, low cardiac output results in a low mixed venous oxygen level [20].

## Diagnosis

**Fig. 2:**
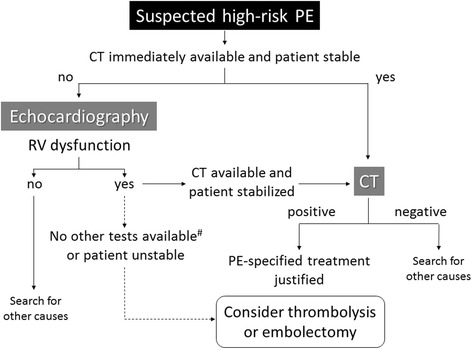
Proposed diagnostic algorithm for patients with suspected high-risk PE. ^#^Apart from the diagnosis of RV dysfunction, bedside transthoracic echocardiography may, in some cases, directly confirm PE by visualizing mobile thrombi in the right heart chambers. Ancillary bedside imaging tests include transesophageal echocardiography, which may detect emboli in the pulmonary artery and its main branches, and bilateral compression venous ultrasonography, which may confirm deep vein thrombosis and thus be of help in emergency management decisions. PE pulmonary embolism, RV right ventricular

The diagnostic strategy [12, 13, 19, 21, 22] for patients with suspected high-risk PE is shown in Fig. 2. Computed tomography (CT) angiography allows not only adequate visualization of the pulmonary thromboemboli down to at least the segmental level but also RV enlargement as an indicator of RV dysfunction. CT venography has been advocated as a simple way to diagnose deep vein thrombosis (DVT) in stable patients with suspected PE because it can be combined with chest CT angiography as a single procedure using only one intravenous injection of contrast dye [23]. If CT angiography is not immediately available or cannot be performed because of hemodynamic instability, bedside transthoracic echocardiography, which will yield evidence of acute pulmonary hypertension and RV dysfunction, is the most useful initial test. In highly unstable patients, the presence of echocardiographic RV dysfunction is sufficient to prompt immediate definitive treatment without further testing. Ancillary bedside imaging tests include transesophageal echocardiography, which may allow direct visualization of thrombi in the pulmonary artery and its main branches, and bilateral compression venous ultrasonography, which may confirm proximal DVT; these techniques may be helpful in emergency management decisions [19].

## Treatment

### Hemodynamic and respiratory support

Acute RV failure with resulting low systemic output is the leading cause of death in patients with high-risk PE. Therefore, supportive treatment is of vital importance in patients with PE who develop shock.

#### Administration of oxygen

Hypoxia is usually reversed with administration of oxygen. When mechanical ventilation is required, care should be taken to limit its adverse hemodynamic effects. In particular, the positive intrathoracic pressure induced by mechanical ventilation may reduce venous return and worsen RV failure in patients with shock; therefore, positive end-expiratory pressure should be applied with caution. Low tidal volumes (approximately 6 ml/kg lean body weight) should be used in an attempt to keep the end-expiratory plateau pressure at < 30 cmH_2_O [19].

#### Modest fluid loading

Experimental studies have shown that aggressive volume loading may worsen RV function by causing mechanical overstretch and/or inducing reflex mechanisms that depress contractility. However, a small clinical study revealed an increase in the cardiac index from 1.7 to 2.1 l/min/m^2^ after infusion of 500 ml of dextran during a 15-min period in normotensive patients with acute PE and a low cardiac index [24]. This finding suggests that a modest fluid challenge may help to increase the cardiac index in patients with PE, a low cardiac index, and normal blood pressure. However, excessive volume loading is not recommended because of the possibility of an increased leftward shift of the interventricular septum [1, 19]. Therefore, the permitted fluid loading volume ranges from 500 to 1000 ml^1^.

#### Vasopressors

Use of vasopressors is often necessary in parallel with (or while waiting for) definitive treatment. Norepinephrine appears to improve RV function via a direct positive inotropic effect while also improving RV coronary perfusion by peripheral vascular alpha receptor stimulation and an increase in systemic blood pressure. No clinical data are available on the effects of norepinephrine in patients with PE, and its use should probably be limited to patients with hypotension [19].

In a small series of patients requiring admission to an intensive care unit for PE, dobutamine increased cardiac output and improved oxygen transport and tissue oxygenation at a constant arterial partial pressure of oxygen. In another study [25] of 10 patients with PE, a low cardiac index, and normal blood pressure, a 35% increase in the cardiac index was observed under intravenous dobutamine infusion at a moderate dosage without significant changes in the heart rate, systemic arterial pressure, or mean pulmonary arterial pressure. Accordingly, the use of dobutamine can be considered for patients with PE, a low cardiac index, and normal blood pressure [19, 21]. However, an increased cardiac index above physiological values may aggravate ventilation–perfusion mismatch by further redistributing flow from partly obstructed to unobstructed vessels. Epinephrine combines the beneficial properties of norepinephrine and dobutamine without the systemic vasodilatory effects of the latter drug. Epinephrine may exert beneficial effects in patients with PE and shock.

#### Inhalation of nitric oxide

Inhalation of nitric oxide improves ventilation–perfusion mismatch in association with selective dilation of the pulmonary artery without systemic vasodilation. It is considered one therapeutic option in patients whose condition is unresponsive to standard treatment [26].

#### Extracorporeal membrane oxygenation

Experimental evidence suggests that extracorporeal membrane oxygenation (ECMO) support can be an effective procedure in patients with PE-induced circulatory collapse. This notion is supported by the results of a series of 10 patients with massive PE requiring ECMO with catheter-based treatment [27]. The mean duration of ECMO was 48 ± 44 h, and the 30-day mortality rate was 30% [27].

### Pharmacological treatment

#### Anticoagulation

Anticoagulant treatment plays a pivotal role in the management of patients with PE. The need for immediate anticoagulation in patients with PE is based on a landmark study [28] that was performed in the 1960s and demonstrated the benefits of unfractionated heparin (UFH) in comparison with no treatment. The efficacy of UFH is attributed to impairment of clot propagation and prevention of recurrent PE. The risk of recurrent PE is highest in the early stages, during which time it is crucial to rapidly achieve a therapeutic level of anticoagulation. An inability to establish a therapeutic activated partial thromboplastin time (aPTT) early in the disease course is associated with a higher rate of recurrence [29].

Because of the high mortality rate in untreated patients, anticoagulant treatment should be considered in patients with suspected PE while awaiting definitive diagnostic confirmation. When high- or intermediate-risk PE is first suspected, patients should receive a bolus of UFH provided that no contraindications to anticoagulation are present.

If intravenous UFH is given, a weight-adjusted regimen of 80 U/kg as a bolus injection followed by infusion at the rate of 18 U/kg/h is preferred to fixed doses of UFH [19, 21, 22]. Subsequent doses of UFH should be adjusted using an aPTT-based nomogram to rapidly reach and maintain aPTT prolongation (1.5–2.5 times control) corresponding to therapeutic heparin levels [19, 21, 22]. The aPTT should be measured 4 to 6 h after the bolus injection and then 3 h after each dose adjustment or once daily when the target therapeutic dose has been reached. Oral anticoagulants can be initiated after hemodynamic stabilization has been achieved. When using warfarin, UFH infusion should be continued until the international normalized ratio has been maintained at therapeutic levels for 2 consecutive days. The UFH infusion can be switched to direct oral anticoagulants; however, direct oral anticoagulants have not been assessed in patients with high-risk PE who have been initially treated with thrombotic therapy. According to an expert comment [30], the introduction of any anticoagulant should be postponed until after the patient has been stabilized with hemodynamic support and after the period of increased bleeding risk related to thrombolytic therapy has passed, which usually lasts 48 to 72 h.

#### Thrombolytic treatment

Thrombolytic treatment of acute PE restores pulmonary perfusion more rapidly than anticoagulation with UFH alone [31, 32]. The early resolution of pulmonary obstruction leads to a prompt reduction in pulmonary artery pressure and resistance, with a concomitant improvement in RV function [32]. In one study, the pulmonary diffusing capacity after 1 year was higher in patients treated with thrombolytic treatment than in those treated with only anticoagulation [33].

The hemodynamic benefits of thrombolysis are confined to the first few days; in survivors, differences are no longer apparent at 1 week after treatment [31]. Accelerated regimens involving administration of tissue plasminogen activator (t-PA) during a 2-h period are preferable to prolonged infusions of first-generation thrombolytic agents during a 12- to 24-h period [34]. Compared with the properties of native t-PA, third-generation bioengineered thrombolytic agents (tenecteplase and monteplase) have a longer half-life, greater clot sensitivity, and more rapid lytic capacity [19, 35, 36]. Monteplase has been approved for acute PE with hemodynamic instability in Japan [35, 36]. Overall, more than 90% of patients appear to respond favorably to thrombolysis as judged by clinical and echocardiographic improvement within 36 h [37]. The greatest benefit is observed when treatment is initiated within 48 h of symptom onset, but thrombolysis can still be useful in patients who have had symptoms for 6 to 14 days [38].

However appealing the rapid resolution of embolic obstruction may be, only one trial has demonstrated a benefit in terms of mortality [39]. However, the results of this small trial of only eight patients should be viewed with caution. All four patients randomized to thrombolytic therapy were treated within 4 h of presentation, whereas those patients randomized to heparin therapy had previously failed to respond to it and developed recurrent PE with severe respiratory failure. A review of randomized trials performed before 2004 indicated that thrombolysis was associated with a significant reduction in mortality or recurrent PE in high-risk patients presenting with hemodynamic instability as compared with anticoagulation (9.4 vs. 19.0%, respectively; odds ratio, 0.45; number needed to treat = 10) [40].

Thrombolytic treatment carries a risk of major bleeding, including intracranial hemorrhage. A meta-analysis of pooled data from trials using various thrombolytic agents and regimens showed an intracranial bleeding rate of 1.46% [41]. In a meta-analysis comparing thrombolysis vs. anticoagulation with UFH alone [42], major bleeding including intracranial or retroperitoneal bleeding, bleeding requiring blood transfusion, or bleeding requiring surgical hemostasis was observed significantly more often in patients undergoing thrombolysis than anticoagulation (13.7 vs. 7.7%, respectively). In the subgroup analysis of that study [42], major bleeding was not significantly increased in patients aged ≤ 65 years (odds ratio, 1.25; 95% confidence interval, 0.50–3.14). However, there was an association with a greater risk of major bleeding in those aged > 65 years (odds ratio, 3.10; 95% confidence interval, 2.10–4.56). Increasing age and the presence of comorbidities including cancer, diabetes, a high prothrombin time–international normalized ratio, or concomitant use of catecholamines have been associated with a higher risk of bleeding complications [43]. In a recent study, a strategy using reduced-dose recombinant t-PA appeared to be safe in patients with hemodynamic instability or massive pulmonary obstruction [44]. In patients with mobile right heart thrombi, the therapeutic benefits of thrombolysis remain controversial [45–47].

Some researchers have proposed that anticoagulation therapy with heparin will prevent the accretion of new fibrin on the thrombus, thereby facilitating lysis by thrombolytic agents and reducing the risk of re-extension after thrombolysis [48]. Unfractionated heparin infusion can be continued during recombinant t-PA infusion.

Absolute contraindications for thrombolysis are active bleeding, ischemic stroke within 2 months, and a history of hemorrhagic stroke. Relative contraindications include a major operation within 10 days, multiple trauma within 2 weeks, neurosurgery or ophthalmologic operations within 1 month, and similar conditions [12]. However, these relative contraindications are also associated with inducible risks for PE. Therefore, thrombolytic therapy may still be appropriate for patients with severe PE complicated by relative contraindications. In patients with confirmed PE as the precipitant of cardiac arrest, thrombolysis is a reasonable emergency treatment option. Thrombolysis may be considered when cardiac arrest is suspected to be caused by PE [49].

### Catheter-directed treatment

Catheter-directed treatment (CDT) can be performed as an alternative to thrombolysis when a patient has absolute contraindications to thrombolysis, as adjunctive therapy when thrombolysis has failed to improve hemodynamics, or as an alternative to surgery if immediate access to cardiopulmonary bypass is unavailable [19]. The objective of CDT is the removal of obstructing thrombi from the main pulmonary arteries to facilitate RV recovery and improve symptoms and survival [50]. For patients with absolute contraindications to thrombolysis, interventional options include thrombus fragmentation with a pigtail or balloon catheter, rheolytic thrombectomy with hydrodynamic catheter devices, and suction thrombectomy with aspiration catheters. Conversely, for patients without absolute contraindications to thrombolysis, catheter-directed thrombolysis or pharmacomechanical thrombolysis are preferred approaches. With respect to thrombus fragmentation, the fact that the cross-sectional area of the distal arterioles is more than four times that of the central circulation and that the volume of the peripheral circulatory bed is about twice that of the pulmonary arteries suggests that the redistribution of large central clots into smaller clots in the peripheral pulmonary arteries may acutely improve cardiopulmonary hemodynamics, with significant increases in the total pulmonary blood flow and RV function [51]. The action of these thrombectomy devices can sometimes be facilitated by softening the thrombotic mass using thrombolytic therapy, which helps to speed up the debulking and fragmentation of the occlusive clots. Fragmentation can also be used as a complement to thrombolytic therapy because fragmentation of a large clot exposes fresh surfaces on which endogenous urokinase and infused thrombolytic drugs can work to further break down the resulting emboli [51]. One review on CDT included 35 nonrandomized studies involving 594 patients [52]. The rate of clinical success, defined as stabilization of hemodynamic parameters, resolution of hypoxia, and survival to discharge, was 87%. The contribution of the mechanical catheter intervention per se to clinical success is unclear because 67% of patients also received adjunctive local thrombolysis. Publication bias probably resulted in underreporting of major complications (reportedly affecting 2% of interventions), which may include death from worsening RV failure, distal embolization, pulmonary artery perforation with lung hemorrhage, systemic bleeding complications, pericardial tamponade, heart block or bradycardia, hemolysis, contrast-induced nephropathy, and puncture-related complications [50]. While anticoagulation with heparin alone has little effect on improvement of RV size and performance within the first 24 to 48 h, the extent of early RV recovery after low-dose catheter-directed thrombolysis appears comparable with that after standard-dose systemic thrombolysis. In a randomized controlled clinical trial of 59 patients with intermediate-risk PE, when compared with treatment by heparin alone, catheter-directed ultrasound-accelerated thrombolysis (administration of 10 mg t-PA per treated lung over 15 h) significantly reduced the subannular RV/LV dimension ratio between baseline and the 24-h follow-up without an increase in bleeding complications [53].

According to a recent guideline [19], CDT should be considered as an alternative to surgical pulmonary embolectomy for patients in whom full-dose systemic thrombolysis is contraindicated or has failed.

### Surgical embolectomy

Traditionally, surgical embolectomy has been reserved for patients with PE who may need cardiopulmonary resuscitation. It is also performed in patients with contraindications or inadequate responses to thrombolysis and in those with patent foramen ovale and intracardiac thrombi [19]. Pulmonary embolectomy is technically a relatively simple operation. ECMO can be helpful in critical situations, ensuring circulation and oxygenation until a definitive diagnosis is obtained [54]. After rapid transfer to the operating room and induction of anesthesia and median sternotomy, normothermic cardiopulmonary bypass should be instituted. Aortic cross-clamping and cardioplegic cardiac arrest should be avoided [55]. With bilateral pulmonary artery incisions, clots can be removed from both pulmonary arteries down to the segmental level under direct vision. Prolonged periods of postoperative cardiopulmonary bypass and weaning may be necessary for recovery of RV function. With a rapid multidisciplinary approach and individualized indications for embolectomy before hemodynamic collapse, perioperative mortality rates of ≤ 6% have been reported [55, 56]. Preoperative thrombolysis increases the risk of bleeding, but it is not an absolute contraindication to surgical embolectomy [57]. The long-term postoperative survival rate, World Health Organization functional class, and quality of life were favorable in published series [54, 58]. Patients presenting with an episode of acute PE superimposed on a history of chronic dyspnea and pulmonary hypertension are likely to develop chronic thromboembolic pulmonary hypertension. These patients should be transferred to an expert center for pulmonary endarterectomy.

### Inferior vena cava filters

In general, inferior vena cava (IVC) filters are indicated in patients with acute PE who have absolute contraindications to anticoagulant drugs and in patients with objectively confirmed recurrent PE despite adequate anticoagulation treatment. Observational studies have suggested that insertion of a venous filter might reduce PE-related mortality rates in the acute phase [59, 60], this benefit possibly coming at the cost of an increased risk of recurrence of venous thromboembolism (VTE) [60]. Although complications associated with permanent IVC filters are common, they are rarely fatal [61]. Overall, early complications, which include insertion-site thrombosis, occur in approximately 10% of patients. Late complications are more frequent and include recurrent DVT in approximately 20% of patients and post-thrombotic syndrome in up to 40% of patients. Occlusion of the IVC affects approximately 22% of patients at 5 years and 33% at 9 years, regardless of the use and duration of anticoagulation [62]. Impermanent IVC filters are classified as temporary or retrievable devices. Temporary filters must be removed within a few days, while retrievable filters can be left in place for longer periods. Impermanent filters should be removed as soon as it is safe to use anticoagulants. The Prévention du Risque d’Embolie Pulmonaire par Interruption Cave II trial enrolled patients with acute symptomatic PE with concomitant DVT and at least one independent risk factor for fatal PE (age of > 75 years, RV dysfunction and/or elevated troponin and/or hypotension, bilateral DVT and/or iliocaval DVT, active cancer, or chronic cardiac or respiratory failure) [63]. The primary end point was fatal and nonfatal PE recurrence at 3 months. The investigators found no significant reduction in the primary end point for patients who received an IVC filter (relative risk with filter, 2.00; 95% confidence interval, 0.51–7.89) [63].

Although some observational data suggest that IVC filter placement in addition to anticoagulation might improve survival in patients with unstable PE or after thrombolytic therapy, controlled data do not support its routine use in patients at high risk of death unless there is a contraindication to anticoagulant therapy [60]. There are no data to support the routine use of venous filters in patients with high-risk PE.

### Treatment algorithm for high-risk PE

**Fig. 3:**
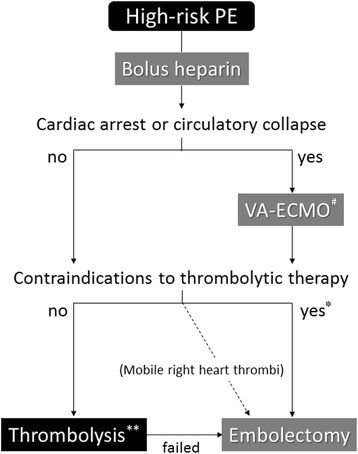
Treatment algorithm for high-risk PE. ^#^Consider ECMO according to hospital equipment and patient condition.*Select appropriate treatment according to hospital equipment and patient condition. **Consider reduced-dose and stepwise thrombolysis for patients in whom the risk of bleeding cannot be ruled out. ECMO extracorporeal membrane oxygenation

An institutional protocol for high-risk PE should be adopted. Figure 3 shows a treatment algorithm for high-risk PE.

## VTE prevention

VTE is a well-recognized life-threatening complication in patients admitted to the intensive care unit (ICU). Patients in the ICU often have multiple thrombotic and bleeding risk factors and should undergo prevention of VTE based on individual assessment of the level of risk. An institution-wide protocol for the prevention of VTE is recommended [64, 65]. The routine use of ultrasonographic screening for DVT is not recommended when thromboprophylactic measures are in place because the detection of asymptomatic DVT may prompt therapeutic anticoagulation that may increase the bleeding risk and has not been proven to reduce significant VTE events. Pharmacological prophylaxis for critically ill patients is effective and is advocated by recent guidelines. Mechanical devices such as intermittent pneumatic compression devices are recommended for patients with contraindications to pharmacological prophylaxis. Generally, pharmacological prophylaxis with low-molecular-weight heparin (LMWH) is recommended over low-dose UFH [64]. Prophylaxis using LMWH and indirect factor Xa inhibitors has stable effects without significant individual differences, and these drugs can be administered subcutaneously once or twice a day without close monitoring. The incidence of adverse drug reactions such as thrombocytopenia and osteopenia is low. In Japan, enoxaparin, a type of LMWH, and fondaparinux, an indirect factor Xa inhibitor, are officially indicated only for patients following orthopedic surgery of a lower limb or abdominal surgery associated with a high risk of development of VTE [21]. Therefore, ICU patients in Japan are prevented by adjusted-dose UFH, which is administered to maintain the aPTT at the upper limit of the normal range. For ICU patients with severe renal insufficiency, the use of low-dose UFH, dalteparin, or reduced-dose enoxaparin is recommended. No study has prospectively evaluated the efficacy and safety of DVT prophylaxis in ICU patients with severe liver dysfunction. Thus, the use of pharmacological prophylaxis in these patients should be carefully balanced against the risk of bleeding. For ICU patients, the routine use of inferior vena cava filters is not recommended for the primary prevention of VTE [64]. When the diagnosis of heparin-induced thrombocytopenia is suspected or confirmed, all forms of heparin must be discontinued and immediate anticoagulation with non-heparin anticoagulants such as argatroban is recommended [64].

.

## Future perspective

Patients with high-risk PE have a potential for circulatory collapse, and thrombolysis is therefore often contraindicated. Physicians should rapidly and properly evaluate patients with PE, formulate a treatment plan, and mobilize the necessary resources to provide the highest level of care. Some centers have recently introduced a formalized system involving a multidisciplinary pulmonary embolism response team to streamline the care of these patients [1, 66]. The team comprises specialists in cardiology, emergency medicine, radiology, cardiovascular surgery, and critical care with an interest in PE. However, how widespread these models have become and whether a multidisciplinary approach to patients with life-threatening PE will be accompanied by improvements in clinical outcomes remain unclear.

## Conclusions

High-risk PE is a life-threatening disorder associated with high mortality and morbidity. Most deaths in patients with shock occur within the first few hours after presentation, and rapid diagnosis and treatment is therefore essential to save patients’ lives. High-risk PE is an indication for thrombolytic therapy but has the potential for circulatory collapse and is therefore often a contraindication to thrombolysis. Large hospitals having an intensive care unit should preemptively establish diagnostic and therapeutic protocols and rehearse multidisciplinary management for patients with high-risk PE.

## Abbreviations

aPTT: Activated partial thromboplastin time
CDT: Catheter-directed treatment
CT: Computed tomography
DVT: Deep vein thrombosis
ECMO: Extracorporeal membrane oxygenation
ICU: Intensive care unit
IVC: Inferior vena cava
LMWH: Low-molecular-weight heparin
LV: Left ventricular
PE: Pulmonary embolism
RV: Right ventricular
t-PA: Tissue plasminogen activator
UFH: Unfractionated heparin
VTE: Venous thromboembolism
